# Community-Onset Extended-Spectrum β-Lactamase–Producing *Enterobacteriaceae* Invasive Infections in Children in a University Hospital in France

**DOI:** 10.1097/MD.0000000000003163

**Published:** 2016-03-25

**Authors:** Julie Toubiana, Sandra Timsit, Agnès Ferroni, Marie Grasseau, Xavier Nassif, Olivier Lortholary, Jean-Ralph Zahar, Martin Chalumeau

**Affiliations:** From the Department of General Pediatrics and Pediatric Infectious Disease (JT, MG, MC); Department of Pediatric Emergency (ST); Department of Microbiology (AF, XN, J-RZ); Department of Infectious Diseases and Tropical Medicine (OL), Necker Enfants-Malades Hospital, Necker-Pasteur Infectious Diseases Center, Université Paris Descartes, IHU Imagine, Paris; and Unité de Prévention et de Lutte contre les Infections Nosocomiales (J-RZ), CHU d’Angers, Université d’Angers, Angers, France.

## Abstract

Limited data is available on pediatric community-onset infections with extended-spectrum β-lactamase-producing *Enterobacteriaceae* (ESBL-PE), but such infections may affect both the efficacy of empiric antibiotic therapy and the rational use of antibiotics.

We retrospectively analyzed data from 2007 to 2012 for all children ≤16 years old with a positive ESBL-PE strain from usually sterile sites within 48 hours of admission in a tertiary hospital in France. We analyzed healthcare- and community-associated infections among community-onset infections. In total, 3612 *Enterobacteriaceae* isolates were collected; the prevalence of ESBL-PE infection increased over the study period, from 2.4% to 5.1% (*P* < 0.001). Among the 90 children with a first community-onset ESBL-PE infection, 58% (n = 52) had a healthcare-associated infection, and 87% of isolates were susceptible to amikacin. As compared with patients with community-associated infections, those with healthcare-associated infections had fewer urinary tract infections (UTIs) (86% vs 97%) and *Escherichia coli* infections (35% vs 84%) and more *Klebsiella pneumoniae* infections (46% vs 8%). Inappropriate empiric treatment was prescribed for 54 patients (64%), but a favorable outcome was observed in 46 of 49 (94%) and 1 of 5 (20%) patients with UTIs and non-UTIs, respectively (*P* < 0.001). Among patients with community-associated infections, 85% had at least 1 risk factor for ESBL-PE infections. In conclusion, the prevalence of community-onset ESBL-PE infections doubled during the study period. These infections mainly occurred among children with healthcare-associated criteria or identified risk factors. Amikacin is an alternative to carbapenems for empiric treatment because most of these infections involved urinary tract and susceptible isolates.

## INTRODUCTION

Community-associated infection caused by extended-spectrum β-lactamase-producing *Enterobacteriaceae* (ESBL-PE) has emerged in many countries worldwide over the past 10 years;^1–5^ the major pathogen involved is ESBL-producing *Escherichia coli*. With a 10-fold increased fecal carriage of ESBL-PE in both adult^6–8^ and children^9^ outpatients, the frequency of community-onset ESBL-producing urinary tract infections (UTIs) in children seemed to increase significantly, reaching 5% in Israel.^10^ The extent of all community-onset invasive infections caused by ESBL-PE and their resistant profile have been poorly studied in children, despite the threat they represent at the patient and population levels. Indeed, ESBL-PE infections are associated with a delay in effective antibiotic therapy and, for bloodstream infections, increased risk of death for both adults and children.^11–15^ However, empiric broad-spectrum antibiotic therapy has serious ecological consequences.^16^

Studies of the prevalence of ESBL-PE in children have mainly focused on fecal carriage in the community,^9,17–19^ and risk factors for community-onset infection caused by ESBL-PE (e.g., presence of comorbidities and recent hospitalization or antibiotic therapy, notably cephalosporins and fluoroquinolones) have been more studied in adults^1,20–37^ than in children.^10,11,29,38–40^ Most studies did not differentiate community- and healthcare-associated infections, the latter now considered almost identical to nosocomial infections.^41^ Indeed, nonhospitalized patients admitted for infection and receiving care at home or in nursing homes tend to have a high rate of bloodstream infections^41–43^ and are often colonized or infected with resistant strains, with a less favorable outcome.^44–46^ Therefore, specifying whether community-onset infection is community- or healthcare-associated would help clinicians evaluate the risk of ESBL-PE community infections and choose the appropriate empiric therapy.

We aimed to study the epidemiology of community-onset ESBL-PE invasive infection in children hospitalized in a tertiary center in France. We examined the prevalence and trends, site of infections, strains involved and their resistance profile, and adequacy of empiric treatment and its relation to patient outcomes, with a special focus on the community and healthcare association of the infection. We also sought to describe the frequency of factors previously reported in the literature and associated with ESBL-PE community-onset infections.

## PATIENTS AND METHODS

### General Methodology

We performed a retrospective observational study in a tertiary care teaching hospital between April 2007 and December 2012. The study was approved by the local ethics committee (no. 2013-09-05). We collected clinical and laboratory data for children ≤16 years old with community-onset invasive ESBL-PE infection who visited the emergency department or were hospitalized. Cases were identified retrospectively by using the microbiological laboratory informatics system. Data for demographic characteristics, medication use, comorbid medical conditions, and current medical history were extracted from the patient medical record.

### Definitions

*Enterobacteriaceae* were identified by using Api 20NE strips (BioMerieux, Marcy-l’Etoile, France) or MALDI-TOF mass spectrometry (Andromas system, Paris, France). ESBL detection and in vitro susceptibility testing involved a double-disk synergy test between clavulanic acid and extended-spectrum cephalosporins (ceftazidime and ceftriaxone) and the disk diffusion method, respectively, as recommended by EUCAST.^47^

Invasive community-onset infection was defined according to the US Centers for Disease Control and Prevention:^48^ clinical symptoms before the patient was admitted to the hospital or within the first 48 hours of hospitalization, with a positive surgical sample (e.g., abdomen, bone, and joint), cerebrospinal fluid (CSF), blood cultures associated with clinical or radiological signs of infection, or urine specimen collected by catheterization (>10^3^ colony forming units/mL) for nontoilet-trained children and clean-catch midstream specimen (>10^4^ colony-forming units/mL) for toilet-trained children. Positive urine samples collected by urine bags were not considered. Infections were categorized as healthcare-associated with the presence of at least one of the criteria proposed by Friedman et al^41^ and otherwise as community-associated infections. Severe sepsis criteria were those defined in the international Pediatric Sepsis consensus Conference.^49^ Relapse of an ESBL-PE infection was defined by a positive sample with the same bacterial species in the same site within 30 days after the infection.

### Statistical Analysis

The prevalence of ESBL-PE strains among *Enterobacteriaceae* involved in community-onset infections was calculated and tested for trends during the study period (Kendall's Tau b). The characteristics of patients with community-onset ESBL-PE invasive infections were described. Clinical and microbiological characteristics of the first community- or healthcare-associated ESBL-PE invasive infection for each child were compared. The prevalence of reported risk factors was studied: gender,^5,20,23,30,34,38^ age,^1,11,20,23,26,28,31,32,50^ country of birth,^25^ travel outside metropolitan France,^23,32^ underlying chronic disease^23,24,26,28,32,34,36,38,50^ and urinary tract disease,^5,10,20,23,25,27,30,39,40^ previous hospitalization.^10,11,20,21,24,29,31,32,35,39,43,50–52^ (excluding hospitalization within the last 3 months before current infection), previous antibiotic therapy,^1,20,21,25–33,36–38,43,50^ known ESBL carriage;^21^ factors included in the definition of healthcare-associated infections were excluded. Their distribution between patients with community- and healthcare-associated invasive infections was compared. SPSS v21 (SPSS Inc., Chicago, IL) was used for analysis. *P* < 0.05 was considered statistically significant.

## RESULTS

### Community-Onset ESBL-PE Prevalence

**FIGURE 1:**
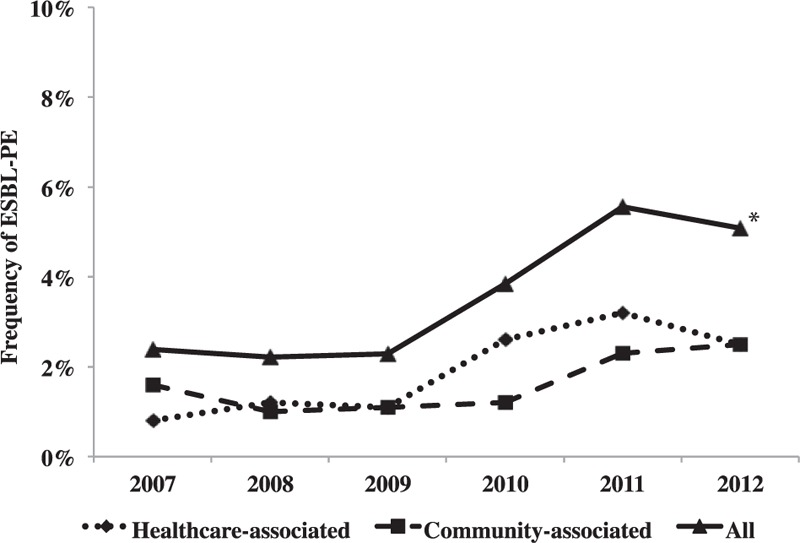
Prevalence of ESBL-PE among *Enterobacteriaceae* strains. Prevalence of ESBL-PE found in community-, healthcare-associated or all (community + healthcare-associated) infections among *Enterobacteriaceae* isolates. ^∗^*P* for trend < 0.001.ESBL-PE = extended spectrum β-lactamases-producing *Enterobacteriaceae.*

During the study period, 3612 *Enterobacteriaceae* isolates were identified from normally sterile samples from children within the first 48 hours of hospitalization: *E coli* (81%), *Proteus* spp. (7%), *Klebsiella* spp. (6%), and *Enterobacter* spp. (3%). Species were isolated from urine (91.8%), peritoneal fluid (3.7%), blood (2.1%), deep abscess (2%), and CSF (0.4%). An ESBL-producing strain was identified in 140 isolates (3.9%), with a significant increase from 2007 (2.4%) to 2012 (5.1%) (*P* < 0.001; Figure 1). The increase was observed for community- and healthcare-associated ESBL-PE infections and mainly concerned 3 species: *E coli* (1.7–3.7%), *K pneumoniae* (21.4–31.3%), and *Enterobacter* spp. (7.7–18.8%). No ESBL-producing *Proteu*s spp. was isolated. ESBL-PE infections were observed in 3.9% (n = 128/3315) of all urinary-infected samples and 4% (n = 12/297) of samples from other sites (12% bloodstream, 1% deep abscesses and peritonitis, and 7% CSF), with a significant increase in ESBL-PE strains during the study period for UTIs from 2007 to 2012 (2.6–5.2%, *P* < 0.001).

### Community-Onset ESBL-PE Infections

For the 3612 isolates, we selected the first community-onset invasive ESBL-PE infection for each patient (Flow-chart in supplementary appendix, Figure S1). Thus, analyses were based on data from 90 patients (median age: 1 years, range 0.7 months to 16 years); 70% were diagnosed in the emergency department, 12% in medical pediatric wards, 11% in surgery pediatric wards, and 7% in intensive care units. Community-associated infections accounted for 42% (n = 38) of ESBL-PE infections and healthcare-associated infections for 58% (n = 52). In total, 44% and 14% of patients had an underlying disease (neurologic or hematologic disorders; on-going cancer; digestive, cardiac or chronic respiratory disease, urinary tract abnormalities) and a premature birth, respectively; both conditions were more frequent in patients with healthcare-associated infections (*P* < 0.005). Healthcare-associated infections were related to previous hospitalization ≤3 months (n = 43, including surgery for n = 15), and/or stay in long-term care facility (n = 9) within the 3 months before the current infection, and/or regular nursing at home for various stomia (n = 19), regular urinary catheterization (n = 12), intravenous therapy on a central venous catheter (n = 9), and/or enteral feeding by nasogastric tube (n = 7).

The most frequent ESBL-PE community-onset invasive infection was febrile UTI for both community- and healthcare-associated infections (91%) (Table 1). Bloodstream infection and meningitis due to ESBL-PE were observed only in healthcare-associated infections*. E coli* was the major *Enterobacteriaceae* causing community-associated infections (84%), and *K pneumoniae* and *Enterobacter cloacae* were strongly associated with healthcare-associated infections (63% vs 8%; *P* < 0.001). All strains were susceptible to imipenem, 79% cefoxitin, 72% piperacillin-tazobactam, and 10% third-generation cephalosporins (Table 1). The only noncarbapenem drug retaining > 80% activity against all isolates was amikacin (87%). Among the 12 community- or healthcare-associated isolates resistant to amikacin, all but one were susceptible to alternative agents to carbapenem: cefoxitin (n = 8), piperacillin-tazobactam (n = 9), and/or ciprofloxacin (n = 5). Susceptible agents available for secondary oral regimens were ciprofloxacin (51%) and trimethoprim (24%). Strains identified in community-associated infections were more frequently susceptible to piperacillin-tazobactam, cefoxitin, and gentamicin than healthcare-associated ones (*P* < 0.05; Table 1).

**TABLE 1:**
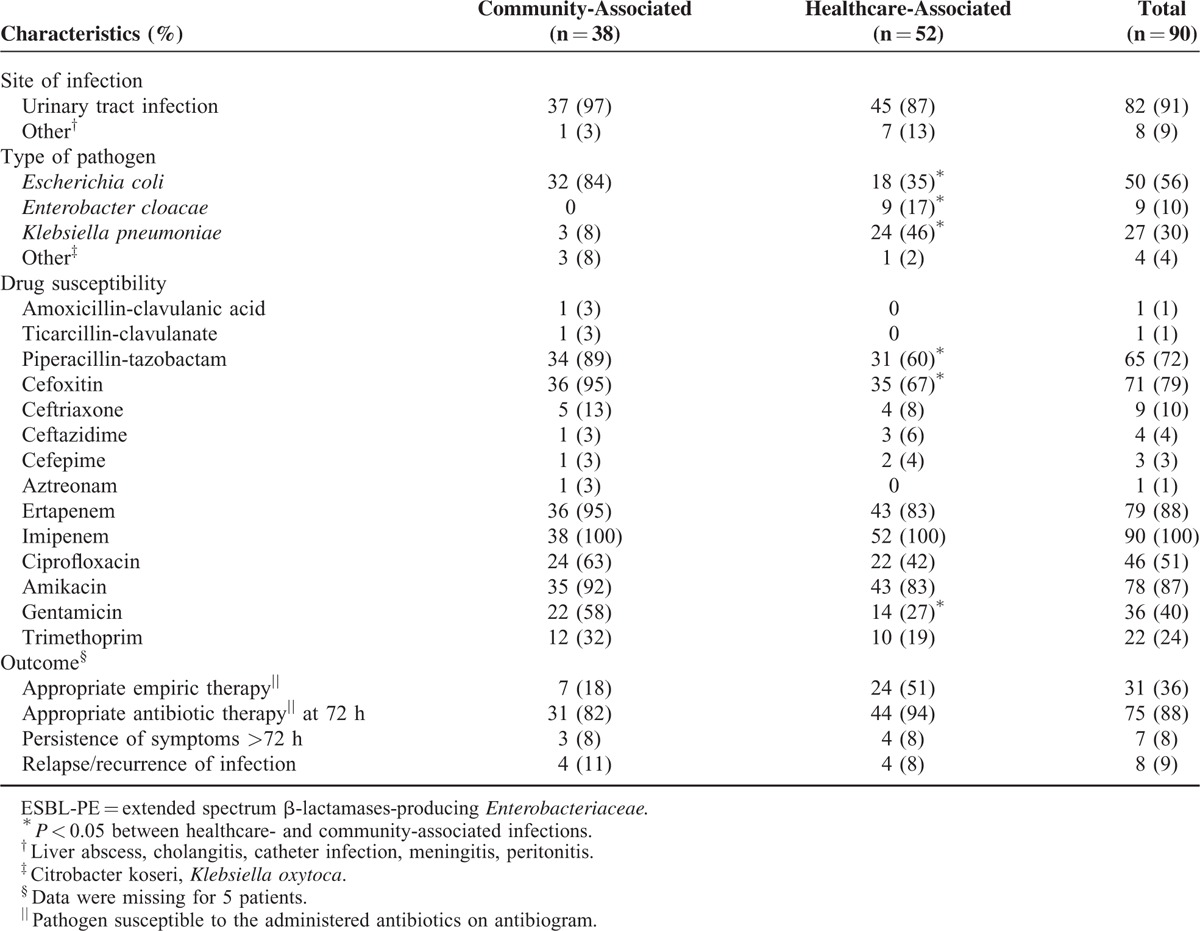
Characteristics of Extended-Spectrum β-Lactamase-Producing *Enterobacteriaceae* (ESBL-PE) Community- and Healthcare-Associated Infections

Severe sepsis tended to be more frequent in healthcare-associated than community-associated episodes (17% vs 5%, *P* = 0.06). No death was attributable to the ESBL-PE infections. Despite inappropriate empiric antibiotic therapy for 54 patients (63%) (Table 1), clinical cure was achieved for 46 of 49 (94%) and 1 of 5 (20%) patients with UTIs and non-UTIs, respectively (*P* < 0.001). The inappropriateness was mostly due to the empirical use of third-generation cephalosporin (83%). Empiric therapy was more appropriate for healthcare- than community-associated infections (51% vs 18%, respectively *P* = 0.001), in relation with a more frequent use of amikacin (45% vs 8%, respectively, *P* < 0.001). At 72 hours after treatment initiation, 88% of patients received appropriate antibiotic therapy (Table 1); 20% of them received alternative therapy to carbapenem. An antibiotic switch was not considered necessary for the other 12% because of clinical cure. Relapse occurred in 9% of patients, reaching 33% for patients who received only third-generation cephalosporin (as compared with 5% for others, *P* = 0.004).

### Prevalence of Risk Factors for Community-onset ESBL-PE Infections

For our patients, factors such as chronic disease, recurrent UTIs, previous hospitalization in intensive care units, history of surgery, antibiotic therapy within the last 6 months, and previously known ESBL-PE fecal carriage were more frequent in healthcare-than community-associated infections (Table 2). Regarding community-associated episodes, previous history of UTIs (29%) or urinary tract abnormalities (40%), previous antibiotic therapy (45%; mainly third-generation cephalosporins), and travel outside of metropolitan France (38%) were the most prevalent risk factors. Among the 33 children with community-associated infections with available data, 28 (85%) had at least 1 risk factor, and 19 (58%) had at least 2 risk factors. Regarding patients with healthcare-associated infections, 49 (95%) had at least 2 risk factors.

**TABLE 2:**
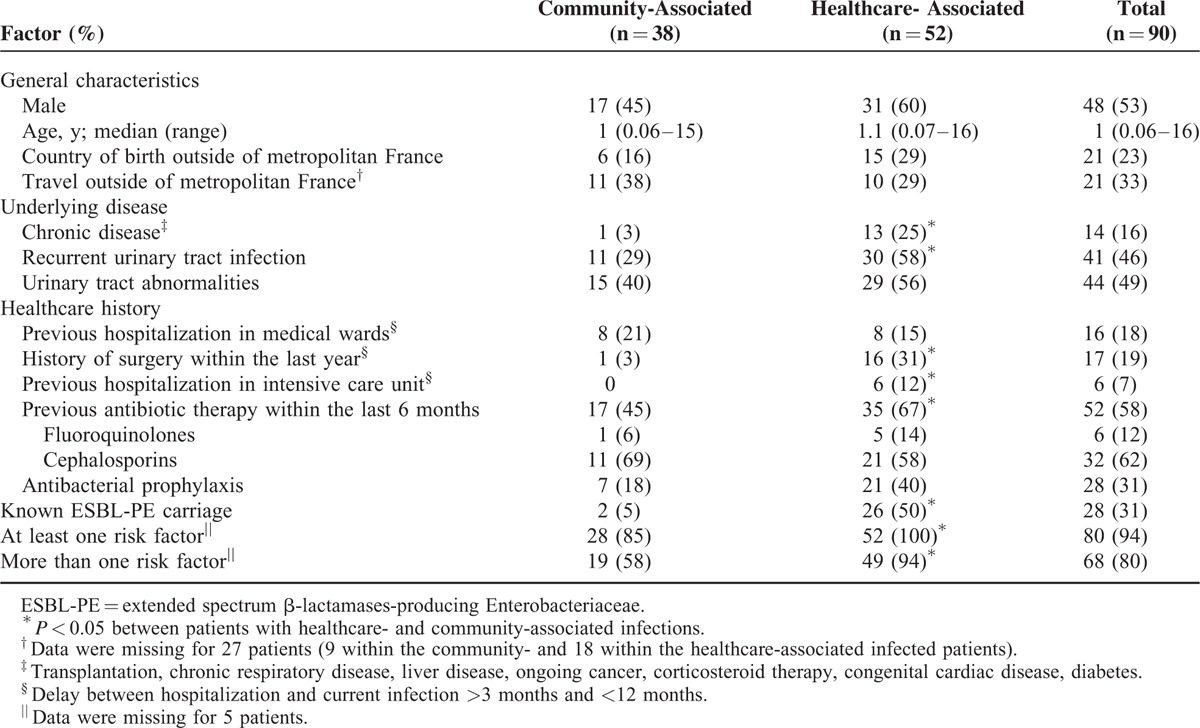
Prevalence of Risk Factors Proposed in the Literature for Children With ESBL-PE Invasive Infections

## DISCUSSION

We report here a significant increase in ESBL-PE among *Enterobacteriaceae* isolated from children with community-onset infections over the 5-year study period in a French university hospital. Similar to previous studies,^11,13,36,38,53^ most infected children (58%) had healthcare-associated infection criteria. Most of the remaining patients had at least 1 risk factor for ESBL-PE infection previously described in the literature. Patients had a favorable outcome despite inappropriate antibiotic prescription only for UTIs, and several alternatives to carbapenems could be considered for treating most of these community-onset ESBL-PE infections.

The prevalence of ESBL-PE among *Enterobacteriaceae* responsible for community-onset invasive infections among children in our hospital in France was still lower than that observed in adults^5,26,35^ but similar to or even higher than the rates observed in recent European pediatric studies.^10,32^ Regarding the marked spread of these resistant isolates in fecal carriage among children without any underlying disease^6,8,9^ and the high rate of ESBL-PE community-onset infections in non-European countries, reaching nearly 10%,^36,38^ pediatricians should be prepared for a continual increase in prevalence of ESBL-PE infection. Among *Enterobacteriaceae* isolated during community-onset infections, *E coli* was the most prevalent, with a proportion of ESBL producers that more than doubled during the last 5 years, as observed in previous studies.^53,54^ The urinary tract was the main site of community-onset infections due to ESBL-producing *E coli*, which may be closely linked to fecal carriage.^55^ Bacteremia might have been underestimated in our study of children as compared to adults^26^ because blood cultures are rarely assessed in children with UTIs. We found positive blood cultures more frequent during healthcare- than community-associated episodes, mainly due to ESBL-producing *K pneumoniae* associated with hepato-biliary or catheter bloodstream infections. These elements suggest that healthcare-associated infections are relatively similar to nosocomial infections in populations for whom the risks are clearly identified.^31,41^ The high rate of healthcare-associated infections (58%), closer to adult findings,^42,43^ was probably related to the particular recruitment of our hospital, with its high-specialized departments and surgical activity.

The increase in ESBL-PE infections we observed highlights the importance of a proper identification of children at high risk. Many studies have identified factors associated with community-onset infections due to ESBL-PE. However, several limitations include unclear definitions of the infection and heterogeneity of the studied groups. By differentiating the healthcare- and community-associated episodes, we revealed the distribution of these risk factors among a homogeneous population of children. Similar to a previous adult study,^42^ we found several factors associated with healthcare-associated infections that are not included in the criteria of Friedman et al.^41^ These variables, such as known fecal carriage of ESBL-PE, previous hospitalization in surgical wards or intensive care unit within the previous 12 months, history of recurrent UTIs or previous antibiotic therapy, should therefore systematically be searched to identify children at high risk of multidrug resistant organisms.^31^ Among patients with community-associated infections, only 5 (15%) had no risk factors. The major risk factors found in these children were urinary tract abnormalities, previous hospitalization or antibiotic therapy, as previously reported.^10,39^ The oral form of third-generation cephalosporin was the main antibiotic prescribed before the episode, the prescription of which is often abused and inappropriate in France.^56^ We acknowledge that, due to the case-only study design, the identification of risk factors associated with ESBL PE community-associated infections was not possible and is part of the limitations of our study.

Inappropriate antibiotic prescription was frequent for our patients and was mostly due to the use of third-generation cephalosporin for community-associated UTIs. The favorable outcome despite inappropriate prescription might be due to spontaneous evolution of the UTI episode and/or the activity of third-generation cephalosporin in ESBL-PE with a low minimal inhibitory concentration (MIC) (because the kidney concentration of the antibiotic is much higher than the MIC),^47^ or both. However, cephalosporins other than cefoxitin must be used carefully because several reports have suggested a high rate of clinical failure with these molecules during ESBL-PE infections even with the MIC of cephalosporin within the susceptible range, especially during extra-renal infection.^11,13^ Antibiotic prescription was more appropriate in healthcare-associated episodes, which were often empirically treated with an aminoglycoside (amikacin) associated with third-generation cephalosporin because of their initial severity and/or known comorbidities. Indeed, 87% of EBLSE-PE isolates were susceptible to amikacin, which suggests that empiric therapy including this aminoglycoside may be of interest for patients with risk factors.^57^ The oral alternatives to carbapenems such as fluroquinolones and cotrimoxazole should be used only after obtaining results of antibiotic susceptibility testing. Unfortunately, the susceptibility rate to these molecules was low, as previously reported,^58^ and prolonged hospitalization was often necessary for intravenous treatment. Cefixim and amoxicillin-clavulanate combination is an interesting oral alternative for treating UTIs after detecting the in vitro interaction between the 2 molecules.^59^ To decrease carbapenem prescription, intravenous treatment with agents such as piperacillin-tazobactam or cephamycins such as cefoxitin was used for 6 patients, as was suggested in several reports.^60,61^

In conclusion, the increase in community-onset ESBL-PE infections in children in our hospital over 5 years was less than that reported for adults. However, the prevalence of these infections is increasing and should be closely monitored. Most of the community-associated ESBL-PE infections were UTIs and the high susceptibility level of the strains to amikacin confirms that amikacin therapy could be considered for UTI treatment when ESBL-PE is suspected, as suggested by international guidelines.^62^ Most patients showed known risk factors, but further investigations are needed to determine the specificity of these risk factors and better define patients with high or low risk of ESBL-PE infections.

## Supplementary Material

Supplemental Digital Content
