# Supplementary figures and images for: Community-Onset Extended-Spectrum β-Lactamase–Producing Enterobacteriaceae Invasive Infections in Children in a University Hospital in France
Source: Medicine (Baltimore). 2016 Mar 25;95(12):e3163. doi: 10.1097/MD.0000000000003163 (PMC4998397; doi:10.1097/MD.0000000000003163)

Supplementary Figure S1. Flow chart for inclusion of infection samples.

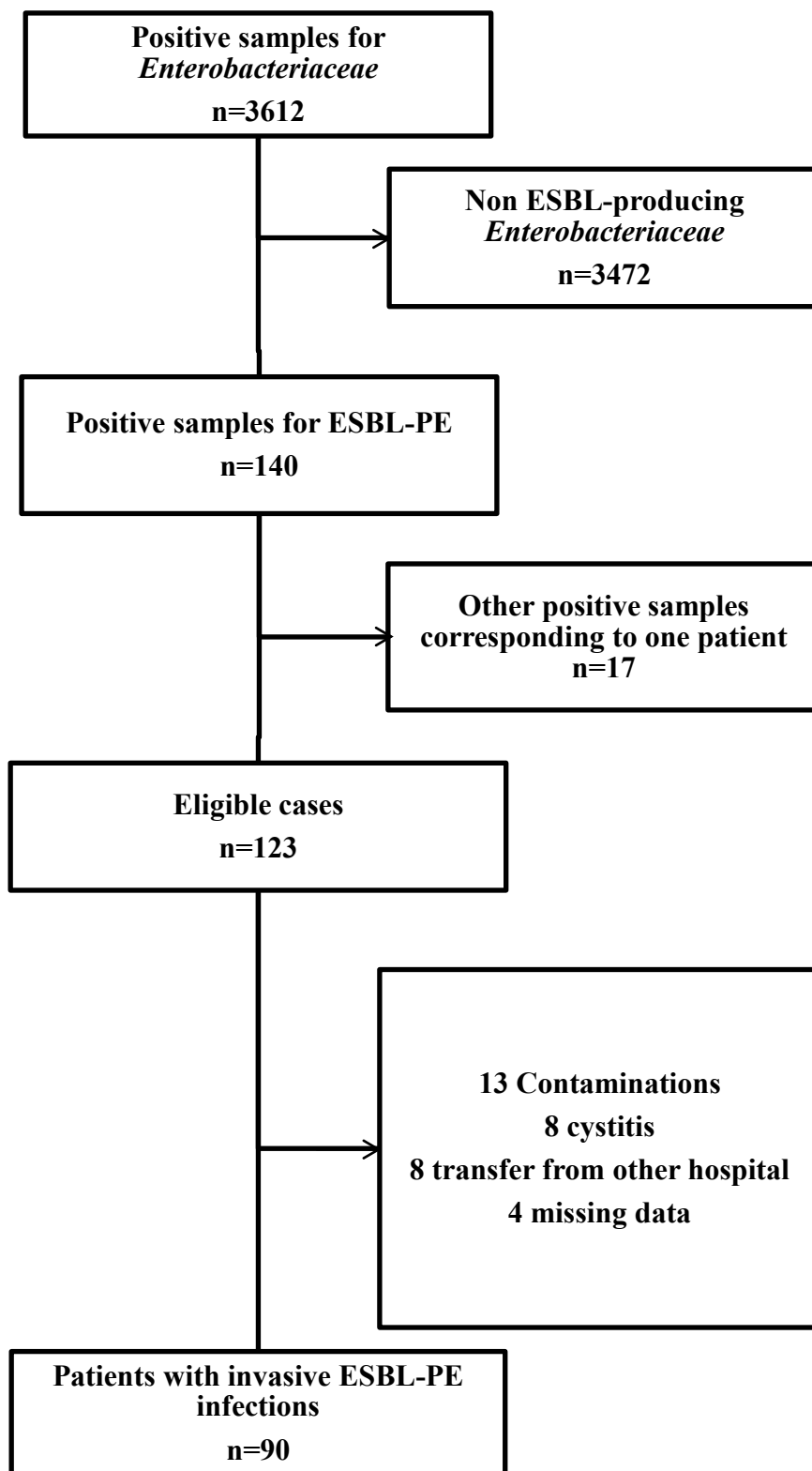

Supplement: Supplemental Digital Content [file medi-95-e3163-s001.pdf]
